# Distribuição Espacial de Mortalidade por Insuficiência Cardíaca no Brasil, 1996-2017

**DOI:** 10.36660/abc.20201325

**Published:** 2021-11-17

**Authors:** Virna Ribeiro Feitosa Cestari, Thiago Santos Garces, George Jó Bezerra Sousa, Thatiana Araújo Maranhão, João David Souza, Maria Lúcia Duarte Pereira, Vera Lúcia Mendes de Paula Pessoa, João Tobias Lima Sales, Raquel Sampaio Florêncio, Lorena Campos de Souza, Glauber Gean de Vasconcelos, Maria Gyslane Vasconcelos Sobral, Lara Lídia Ventura Damasceno, Thereza Maria Magalhães Moreira

**Affiliations:** 1 Universidade Estadual do Ceará Programa de Pós-Graduação Cuidados Clínicos em Enfermagem e Saúde Fortaleza CE Brasil Universidade Estadual do Ceará - Programa de Pós-Graduação Cuidados Clínicos em Enfermagem e Saúde , Fortaleza , CE – Brasil; 2 Universidade Estadual do Piauí Teresina Piauí Brasil Universidade Estadual do Piauí – Enfermagem, Teresina , Piauí – Brasil; 3 Hospital de Messejana Dr. Carlos Alberto Studart Gomes Fortaleza CE Brasil Hospital de Messejana Dr. Carlos Alberto Studart Gomes , Fortaleza , CE – Brasil l; 4 Universidade Estadual do Ceará Centro de Educação Fortaleza CE Brasil Universidade Estadual do Ceará - Centro de Educação , Fortaleza , CE – Brasil

**Keywords:** Insuficiência Cardíaca, Análise espacial, Estudos ecológicos, Epidemiologia

## Abstract

**Fundamento:**

Insuficiência cardíaca (IC) é uma das principais causas de mortalidade e morbidade no mundo, e está associada ao alto uso de recursos e custos com saúde. No Brasil, a prevalência de IC é de aproximadamente 2 milhões de pacientes, e sua incidência é de aproximadamente 240.000 novos casos por ano.

**Objetivo:**

A investigação objetivou analisar a tendência espaço-temporal da mortalidade causada por IC no Brasil, de 1996 a 2017.

**Métodos:**

Este é um estudo ecológico desenvolvido com dados secundários sobre mortalidade por IC no Brasil. Durante o período, 1.242.014 casos de morte causada por IC foram analisados. A existência da autocorrelação espacial de casos foi calculada utilizando o Índice de Moran Global (IMG) e, quando significativo, o Índice de Moran Local, considerando p <0,05. O risco relativo dos grupos foi calculado.

**Resultados:**

A taxa de mortalidade causada por IC foi diversificada em regiões brasileiras, com ênfase no sul, sudeste e nordeste. O IMG indicou autocorrelação espacial positiva (p=0,01) em todos os períodos. Cidades localizadas no sul, sudeste, nordeste e centro-oeste mostraram maior risco relativo para mortalidade causada por IC, e a maioria das cidades do norte foi classificada como um fator protetivo contra esta causa de morte.

**Conclusões:**

O estudo demonstrou declínio nas taxas de mortalidade no território nacional. A maior concentração de taxas de mortalidade está nas regiões norte e nordeste, enfatizando as áreas prioritárias de vulnerabilidade no planejamento e estratégias de controle de serviços de saúde.

## Introdução

A insuficiência cardíaca (IC) é uma das principais causas de mortalidade e morbidade no mundo, e está associada a um alto uso de recursos e despesas com saúde. Nos Estados Unidos, o número estimado de pacientes com IC com ≥ 20 anos aumentou de 5,7 milhões, em 2009-2012, para 6,2 milhões, em 2013-2016,^1 , 2^ e estima-se que afeta 26 milhões de pessoas no mundo. Sua prevalência tem crescido rapidamente devido ao envelhecimento da população e melhor assistência e tratamento.^3 , 4^

No Brasil, a prevalência de IC é de aproximadamente 2 milhões de pacientes, e sua incidência é de 240.000 novos casos por ano. Este país tem o maior sistema universal de saúde do mundo; além disso, caracteriza-se pela intensa mistura racial, desigualdades sociais e tradições culturais que podem afetar a história natural da IC.^5^ Observou-se que as taxas de morbidade e mortalidade relacionadas à IC no Brasil são muito maiores do que aquelas observadas em países desenvolvidos, mesmo quando ajustadas por região, leitos hospitalares e tipo de instituição.^6^

Diversas regiões do Brasil demonstraram grande variação na qualidade do cuidado relacionado a condições cardiovasculares de alta carga econômica, como a IC. Esses registros são insuficientes, e a falta de terapias melhores é mais crítica em instituições públicas não-acadêmicas nas regiões mais vulneráveis do Brasil.^7^

A mortalidade causada pela IC está constantemente associada aos indicadores individuais, sociais, econômicos e de serviços de saúde.^8^ Então, é importante ter um conhecimento aprofundado sobre a distribuição espacial da mortalidade causada pela IC, assim como grande conhecimento sobre as áreas de maior vulnerabilidade. A análise espacial dos casos de mortalidade por IC no território nacional é essencial para a identificação de demandas específicas e localidades prioritárias e, assim, para o planejamento de intervenções que podem reduzir a mortalidade causada por esta doença.

Não há estudos nacionais e internacionais sobre a distribuição e análise espacial de taxas de mortalidade de IC no território nacional, denotando uma lacuna de conhecimento.

## Objetivo

Esta investigação objetivou analisar a tendência espaço-temporal da mortalidade causada por IC no Brasil de 1996 a 2017.

## Métodos

### Desenho

Este é um estudo ecológico que incluiu todas as regiões e cidades do Brasil como unidades de análise. O Brasil tem uma população de 211.389.487 habitantes distribuídos em 5.570 cidades, de acordo com o Instituto Brasileiro de Geografia e Estatística (IBGE) (2020).

**Figura 1:**
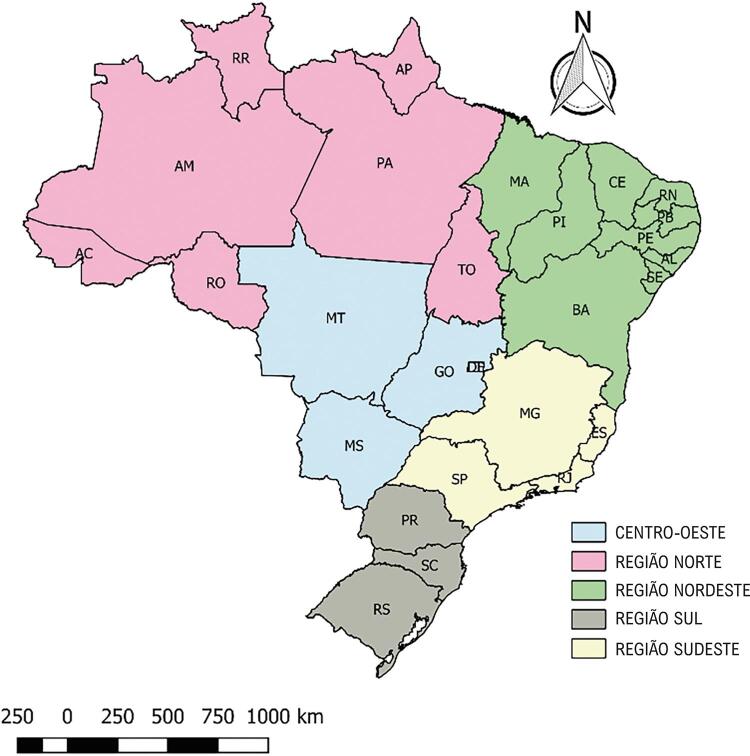
– Regiões e estados brasileiros.

O país é dividido em cinco grandes regiões, subdivididas em 26 estados e o Distrito Federal: sete estados no norte – Acre (AC), Amapá (AP), Amazonas (AM), Pará (PA), Rondônia (RO), Roraima (RR) e Tocantins (TO); nove estados no nordeste – Alagoas (AL), Bahia (BA), Ceará (CE), Maranhão (MA), Paraíba (PB), Pernambuco (PE), Piauí (PI), Rio Grande do Norte (RN) e Sergipe (SE); quatro estados no sudeste – Espírito Santo (ES), Minas Gerais (MG), Rio de Janeiro (RJ) e São Paulo (SP); três estados no sul – Paraná (PR), Rio Grande do Sul (RS) e Santa Catarina (SC); três estados no centro-oeste – Goiás (GO), Mato Grosso (MT), Mato Grosso do Sul (MS); e o Distrito Federal (DF) ( Figura 1 ).

### Cenário

Os dados foram obtidos do site do Sistema Único de Saúde (DATASUS), nas seções Estatísticas Vitais (mortalidade – 1996 a 2017, pelo CID-10), e Indicadores de Saúde pactuados (www.datasus.com.br). Esses dados foram coletados em julho de 2019. A população usada para calcular a taxa de mortalidade por 100 mil habitantes e a base cartográfica digital foram obtidas do IBGE.

### Critérios de inclusão

Todos os dados secundários sobre mortalidade por IC no país foram usados (classificação I50 no CID-10), diagnosticados de janeiro de 1996 a dezembro de 2017, e registrados no Sistema de Informações sobre Mortalidade (SIM).

### Análise

Após identificar o número de mortes causadas por IC nas cidades, a taxa de mortalidade foi calculada. Usamos como numerador o número total de mortes identificadas a cada ano pela população residente no mesmo ano, para cada um dos anos observados, de 1996 a 2017. Então, o resultado foi multiplicado pela constante 100 mil.

Analisamos o padrão temporal da mortalidade por IC no Brasil em geral e por região. Os dados organizados em tabelas, no Microsoft Excel, foram transferidos para o software Jointpoint Regression, versão 4.6.0.0. Com este programa, é possível fazer uma análise de série temporal dos mais diversos problemas de saúde. Também realiza análises lineares segmentadas (também conhecidas como pontos de inflexão, ou *jointpoints* ), calculando a transformação logarítmica dos valores.

O programa trabalha com a hipótese nula de que um segmento de uma linha pode explicar possíveis variações ao longo dos anos, e que a alternativa a este processo seria a inclusão de pontos de inflexão no período, com consequente mudança na curva do segmento linear. O programa calcula a variação percentual anual (VPA), com intervalo de confiança de 95% (IC95%).

A interpretação dada a esses valores é que uma VPA positiva indica tendência crescente, enquanto a negativa indica tendência decrescente. Além disso, ao final do período, é possível calcular como as mudanças durante os períodos se comportam usando a variação percentual anual média (VPAM), cuja interpretação é semelhante à VPA, mas, neste caso, englobando todo o espaço de tempo.

De acordo com este princípio, este estudo trabalhou com a hipótese da nulidade de que uma simples tendência linear poderia expressar a variação da taxa de mortalidade causada por IC no Brasil, e que, como alternativa, pontos deveriam ser incluídos no modelo com mudança nos segmentos lineares. Um nível de significância de 5% foi estabelecido para testar as hipóteses de VPA e VPAM na série. Para ambos os casos, considerou-se significativo quando o modelo tinha p<0,05 ou IC95%, inteiramente positivo ou negativo.

Os anos analisados foram divididos em quatro períodos: A (1996-2001), B (2002-2007), C (2008-2012), e D (2013-2017). Essas taxas brutas foram suavizadas por meio do método Bayesiano empírico para reduzir a instabilidade. Este método considera não só o número de mortes por cidade, mas as pesa com as cidades vizinhas por meio de uma matriz de proximidade espacial. Para calcular esta matriz, o critério de contiguidade foi usado considerando valor 1 para cidades vizinhas, e 0 para as não-vizinhas.^9^

Após uma análise espacial descritiva, a presença da dependência espacial global foi avaliada utilizando o Índice de Moran Global (IMG) sobre indicadores brutos. O método identifica a autocorrelação espacial e varia de -1 a +1, segundo o qual valores próximos a zero indicam a ausência de dependência espacial, considerando p<0,05 como significante. Adicionalmente, o Indicador Local de Associação Espacial (LISA) foi avaliado pelo Índice de Moran Local, que verifica o valor da cidade e das vizinhas para identificar os padrões espaciais do fenômeno estudado.^10^

O Índice de Moran Local gera o diagrama de espalhamento de Moran, que consiste de quatro quadrantes: alto-alto (cidades com altas taxas e rodeadas por outras com altas taxas), baixo-baixo (cidades com baixas taxas e rodeadas por outras com baixas taxas), alto-baixo (cidades com altas taxas rodeadas por outras com baixas taxas), e baixo-alto (cidades com baixas taxas rodeadas por outras com altas taxas), considerando p<0,05 como significativo. As categorias alto-alto e baixo-baixo representam áreas de concordância, e as categorias alto-baixo e baixo-alto indicam áreas de transição epidemiológica.^10^

O risco relativo dos grupos foi calculado por meio da técnica espacial de varredura, chamada de estatística de varredura. Foi utilizado para identificar áreas de risco e proteção para a mortalidade por IC. Para este fim, o risco relativo (RR) de cada cidade para mortalidade foi calculado, e a presença de grupos de morte espacial foi identificada.

A análise estatística foi realizada utilizando o software Stata, versão 12.0. A regressão de pontos de inflexão foi realizada com o software Jointpoint Regression, versão 4.7.0.0. A estatística espacial foi realizada com o TerraView 4.2.2. A análise espacial de varredura foi realizada com a ajuda do software SaTScan 9.6. Todos os mapas foram criados com o QGIS 2.4.17.

### Considerações éticas

Este estudo dispensou a aprovação prévia dos Comitês de Ética e Pesquisa, já que a base de dados estava disponível gratuitamente na internet pelo Governo Brasileiro. É importante enfatizar que não há informações de identificação, como nome ou endereço, na base de dados. Apesar de os pesquisadores não precisarem de aprovação prévia, eles declararam seu compromisso ético ao lidar com os dados, análise e publicação, como estabelecido pela resolução 466/12, do Conselho Nacional de Pesquisa. A pesquisa foi reportada no STROBE (Strengthening the Reporting of Observational studies in Epidemiology Statement).

## Resultados

Durante o período de 21 anos, 1.242.014 mortes foram notificadas por IC. Os resultados da análise da tendência temporal mostraram redução significativa de 2,3% (IC95%: -2,3 – -2,7) na taxa de mortalidade em todos os territórios brasileiros. Ao considerar as dimensões continentais do país, é necessário investigar essas tendências em áreas regionais. Quatro das cinco regiões mostraram redução significativa nas taxas de mortalidade, destacando uma redução mínima nas taxas no nordeste (VPAM: -0,7; IC95%: -0,9 – -0,4). Por outro lado, o sul aumentou sua taxa de mortalidade em 2,9% (IC95%: 2,0 – 3,9) ( Tabela 1 ).

**Tabela 1:** – Tendências temporais da mortalidade por insuficiência cardíaca no Brasil e regiões. Fortaleza, CE, Brasil, 2019

Regiões Do país	*VPA1	^‡^PI	*VPA2	^‡^IP	^*^VPA3	^‡^PI	†VPAM
			
	(§ IC95%)		(§ IC95%)		(§ IC95%))		(§ IC95%))
Brasil	-6,3 **//** (-7,7; -1,8)	2001	-1,1 **//** (-1,3; -0,7)	2017			-2,3 **//** (-2,3; -2,7)
Norte	-1,3 (-11,3; 2,4)	2017					-1,3 **//** (-1,7; -0,9)
Nordeste	- 0,7 **//** (-7,1; -1,5)	2017	9,4 **//** (6,6; 12,3)	2011	-2,8 (-4,2; -1,4)		-0,7 **//** (-0,9; -0,4)
Sudeste	-7,7 (-8,7; -6,7)	2002	-0,8 (-1,2; -0,3)	2014	-2,4 **//** ( -0,9; -5,8)	2017	-4,0 **//** (-5,5; -2,4)
Centro-oeste	-6,2 **//** (-10,2; -2,1)	2000	-2,3 **//** (-3,0; -1,7)	2015	-3,3 ( -11,5; 3,8)	2017	-4,0 **//** (-5,5; -2,4)
Sul	-7,9 **//** ( -13,0; -2,5)	1998	37 **//** (28,9; 45,5)	2001	-1,1 **//** (-1,3; -0,8)	2017	2,9 **//** (2,0; 3,9)

*VPA: Variação Percentual Anual; **†** VPAM: Variação Percentual Anual Média; **‡** PI: Ponto de Inflexão; **§** IC95%: Intervalo de confiança de 95%; **//** p = < 0,05.

**Figura 2:**
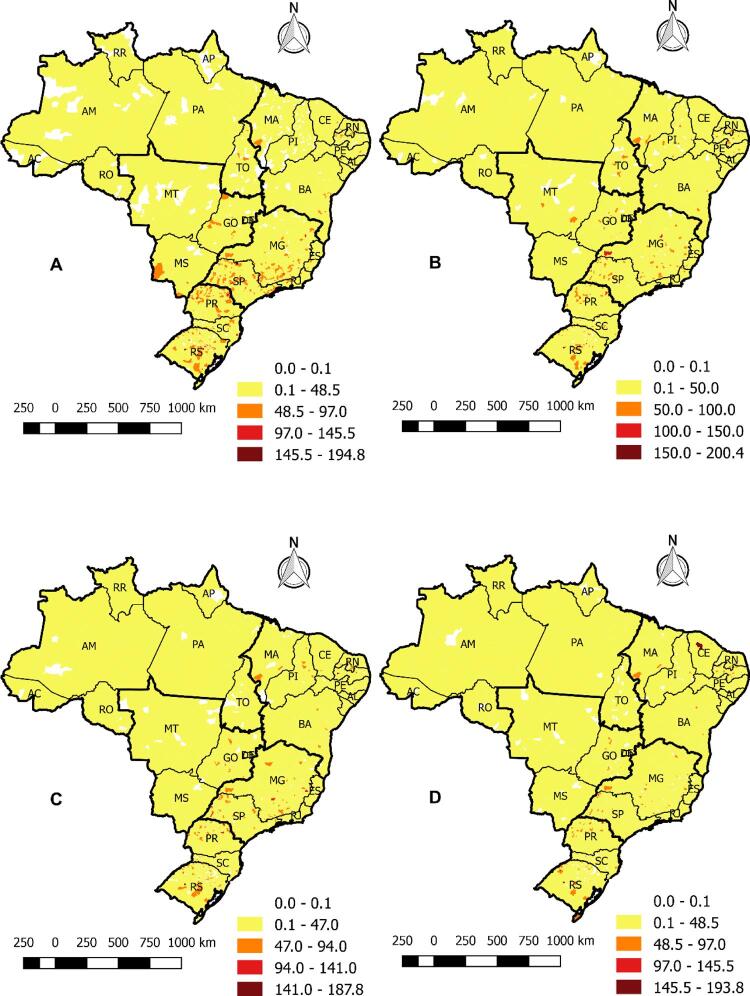
– Mapas das taxas de mortalidade bruta por insuficiência cardíaca no Brasil, nos períodos A (1996-2001), B (2002-2007), C (2008-2012), D (2013-2017), por cidade e por 100 mil habitantes. Fortaleza, CE, Brasil, 2019.

Em relação à análise espacial dos 5.570 municípios, os mapas das Figuras 2 demonstram que as taxas brutas de mortalidade variaram de 0 a 200 mortes por 100 mil habitantes. Nas áreas em vermelho, é possível identificar, no período A (1996-2001), que altas taxas de mortalidade estavam focadas nas cidades do sul e do sudeste. Os períodos B (2002-2007) e C (2008-2012) demonstraram que, ao longo dos anos, as taxas se espalharam para o nordeste e o centro-oeste, mas mantiveram altas concentrações no sul (resultados semelhantes à análise série temporal). Nos últimos anos, como demonstrado no período D (2013-2017), é possível destacar que as cidades do norte não apresentaram altas taxas de mortalidade.

**Figura 3:**
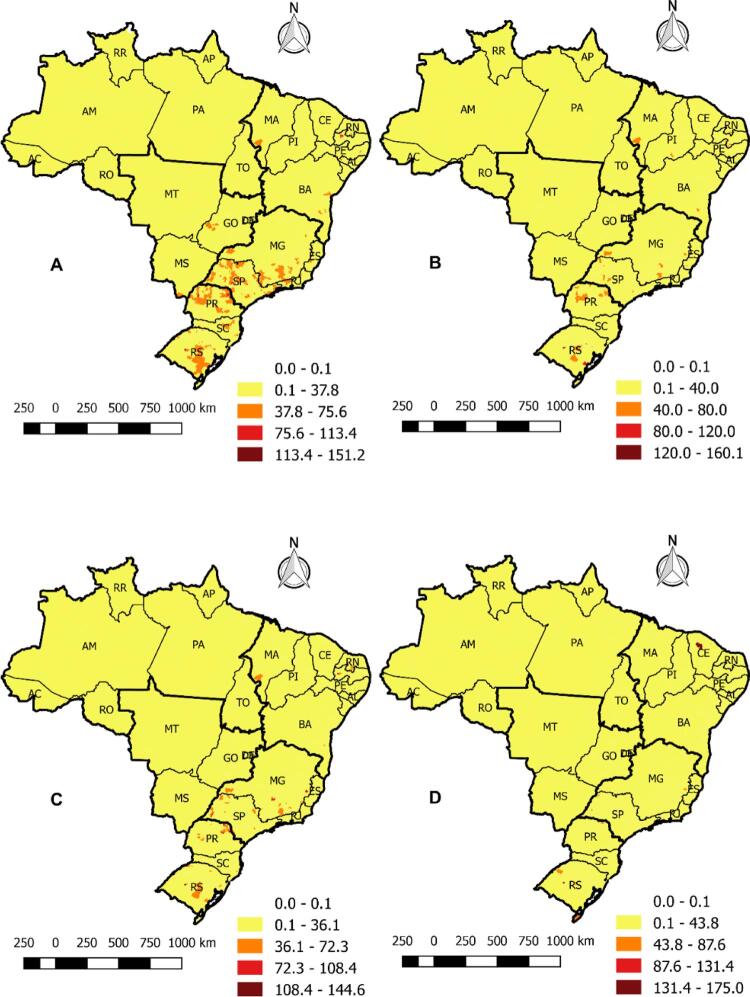
– Mapas das taxas e mortalidade devido à insuficiência cardíaca no Brasil suavizadas pelo método Bayesiano empírico nos períodos A (1996-2001), B (2002-2007), C (2008-2012) e D (2013-2017), por cidade e por 100 mil habitantes. Fortaleza, CE, Brasil, 2019.

Na Figura 3 , as taxas foram suavizadas pelo método Bayesiano empírico e demonstraram que, no período A (1996-2001), a mortalidade estava principalmente concentrada nas cidades do sul; porém, variaram ao longo dos anos. O período B (2002-2007) mostrou que o sudeste apresentou altas taxas de mortalidade, principalmente em cidades localizadas nos estados de São Paulo e Minas Gerais. Nos períodos C (2008-2012) e D (2013-2017), foi possível identificar uma concentração alta de mortes no nordeste, principalmente no Piauí, Bahia, Rio Grande do Norte e Ceará.

Em relação à análise de autocorrelação espacial, o IMG do período A (1996-2001) foi 0,41 (p=0,01); do período B (2002-2007), 0,28 (p=0,01); do período C (2008-2012), de 0,29 (p=0,01); e o IMG no período D (2013-2017) foi de 0,31 (p=0,01). Esses resultados indicam autocorrelação espacial positiva.

**Figura 4:**
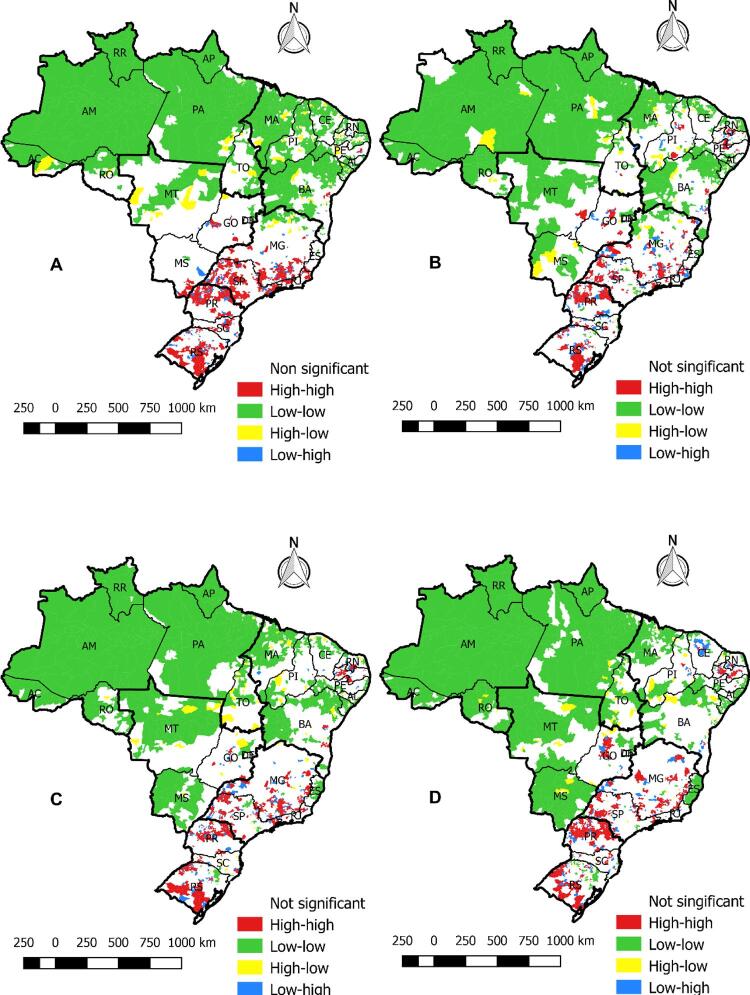
– Mapas da autocorrelação espacial de Moran sobre a mortalidade por insuficiência cardíaca no Brasil nos períodos A (1996-2001), B (2002-2007), C (2008-2012) e D (2013-2017). Fortaleza, CE, Brasil, 2019.

Já que a dependência espacial foi verificada em todas as áreas do estudo pelo IMG, utilizamos o Índice de Moran Local (IML). Os grupos espaciais corroboram os resultados nos mapas locais do método Bayesiano empírico em todos os períodos. Confirmaram que índices alto-alto estão mais concentrados no sul e se espalharam para as cidades do nordeste ao longo dos anos. O índice baixo-baixo esteve concentrado em cidades do norte e centro-oeste ( Figura 4 ).

**Figura 5:**
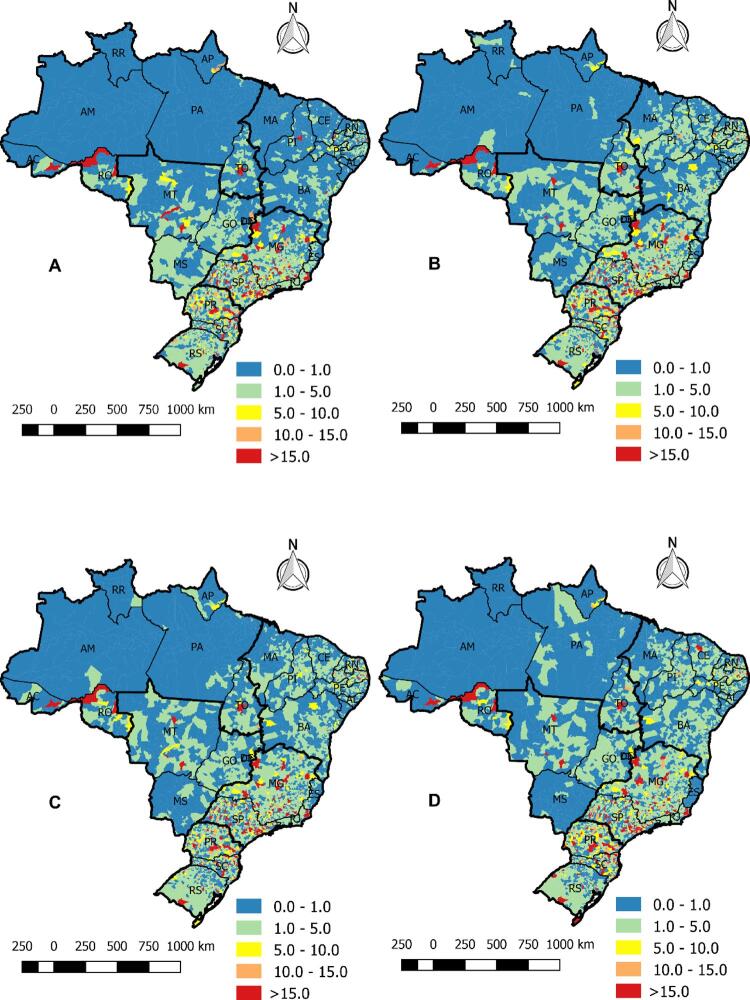
– Risco relativo de todas as cidades brasileiras para mortalidade por insuficiência cardíaca nos períodos A (1996-2001), B (2002-2007), C (2008-2012) e D (2013-2017). Fortaleza, CE, Brasil, 2019.

Na Figura 5 , a estatística espacial de varredura confirmou outras análises e identificou que o maior risco de mortalidade por IC estava no sul no período A (1996-2001). Este padrão avançou em outros períodos, sendo possível encontrar RR alto no centro-oeste.

Porém, o ultimo período também demonstrou altos riscos no norte e nordeste. No último período (2013-2017), quase todas as cidades do sul, sudeste, nordeste e centro-oeste tinham algum risco de mortalidade causada por IC. No norte, as cidades nos estados do Pará e Amazonas tinham pouco risco de mortalidade, com RR<1 (em azul); somente algumas cidades de Roraima e do Acre tinham grupos com RR >15 (em vermelho).

## Discussão

Este é o primeiro estudo que avalia a distribuição espacial de mortalidade por IC no Brasil. Inicialmente, as cidades do sul tinham maior taxa de mortalidade. Este padrão de notificação caracterizava contextos operacionais de “silêncio epidemiológico” em algumas áreas desde 2001. Desde 2002, as taxas de mortalidade aumentaram nas cidades do sudeste, nordeste e centro-oeste. As cidades no norte, por outro lado, mostraram taxas baixas de mortalidade, revelando a subnotificação de casos.

É importante observar que as cidades nas regiões brasileiras com as maiores taxas de mortalidade são as maiores em tamanho da população, e as que têm mais pessoas idosas. Questionários nacionais e internacionais demonstraram que a IC afeta principalmente indivíduos com mais de 65 anos.^11 , 12^ A prevalência crescente de IC está diretamente relacionada às comorbidades mais comuns do envelhecimento.^13^ Além disso, a idade avançada é um fator de risco para o autocuidado, pouca aderência ao tratamento e manejo da doença.^14 - 16^

A doença prevalece entre os mais velhos, mas uma grande porcentagem também é percebida na população adulta.^17^ Em uma análise de tendências temporais específicas por idade, sexo e raça considerando taxas de morbidade e mortalidade relacionadas à IC entre 1991 e 2015, nos Estados Unidos, Vasan, Zuo e Kalesan^18^ observaram um aumento nas taxas de hospitalização de pessoas com a doença na faixa etária dos 30 aos 49 anos. Este fato pode estar relacionado a um estilo de vida inadequado, expondo os indivíduos a diversos fatores de risco para a doença, como o ato de fumar, consumo de álcool, falta de atividade física e comorbidades, com ênfase na hipertensão e diabetes.

Poffo et al.^19^ atribuíram o número de casos de IC em adultos à manifestação precoce da doença cardíaca, tratamentos menos efetivos para doenças que levam à IC, e à não-aderência ao tratamento recomendado. Assim, a prevenção se destaca como arma importante contra a IC.^17 , 18 , 20^ A alta taxa de mortalidade em cidades na região sudeste pode estar associada à raça/etnia de pessoas afetadas pela IC, com maior prevalência entre os brancos. Enquanto a doença prevalece nesta população, a sobrevivência é maior entre os negros (7,5% vs. 2,2%; p = 0,01),^19^ hispânicos e asiáticos.^21^

O motivo para tal disparidade é complexo. O efeito da raça no prognóstico da IC não é claro, porque diferentes estudos trazem resultados divergentes. A raça não demonstrou associação com a ocorrência de complicações e readmissões hospitalares entre os australianos com IC.^22^ Husaini et al.,^23^ em estudo comparando raça/etnia e taxas de internação, descobriram que as taxas de internação para indivíduos negros permaneceu mais alta em comparação aos brancos e hispânicos.

Em um estudo norte-americano, em um total de 7.878 pacientes com IC, 35,8% eram negros. Entre eles, condições como hipertensão, diabetes e doenças cerebrovasculares prevaleceram; porém, a taxa de mortalidade foi mais baixa quando comparados a indivíduos brancos.^24^ Goyal et al.^25^ analisaram 1.889.608 internações de pacientes com IC e descobriram que outras comorbidades, como anemia e doença renal crônica, foram mais comuns entre os negros, mas este grupo apresentou menor mortalidade hospitalar quando comparado aos brancos.

A divergência na literatura reforça a necessidade de mais pesquisas sobre a associação entre raça/etnia e a taxa de mortalidade devido à IC, assim como as principais causas de readmissão. Alguns achados podem ajudar na implementação de programas preventivos para hipertensão e diabetes entre as minorias, o que pode reduzir a subsequente hospitalização por IC nesses pacientes.

A mudança nos padrões epidemiológicos da doença no país também foi gerada como resultado da cobertura dos serviços de saúde. A saúde no Brasil é guiada por uma clara definição de territórios e das pessoas sob a responsabilidade de cada equipe de saúde. As melhorias no acesso, qualidade e infraestrutura do serviço de saúde e a cobertura de cidades brasileiras são preocupações dos administradores da saúde.

É necessário ter em mente que há diferenças regionais entre as condições de acesso a instituições de saúde e a assistência oferecida no Brasil. As diferenças no fornecimento podem ser observadas até dentro da mesma cidade, entre cidades e entre estados da federação, o que leva à manutenção de uma distribuição desigual de fornecimento e acesso de serviços.^26^

A complexidade da IC requer conhecimento de diferentes profissionais que trabalham em diferentes níveis da assistência à saúde. Assim, é importante articular esses níveis para promover saúde, qualidade no atendimento e satisfação do usuário. Os fornecedores da saúde e os pacientes devem assumir alguma responsabilidade que vá além do cuidado mecânico e tradicional. É necessário empoderar e capacitar o indivíduo para que ele se proteja de situações de vulnerabilidade, assim reduzindo as disparidades regionais.

O aumento nas taxas de mortalidade devido à IC nas cidades do norte e nordeste pode ser explicado pela baixa qualidade de vida e fatores estressantes, que são os principais agentes para o aumento na hospitalização. As internações ocorrem não só por conta da IC, mas também por outras comorbidades.^19 , 27^

As regiões brasileiras com o maior número de internações em 2017 foram o sudeste, seguido do sul e nordeste e, em terceiro lugar, o centro-oeste e o norte.^28^ Iniciativas isoladas sugerem a existência de diferenças regionais significativas em várias características de pacientes hospitalizados com IC no Brasil,^17^ embora essas comparações sejam metodologicamente limitadas por desenhos e critérios de inclusão normalmente divergentes.

Apesar dos grandes avanços na ciência e na tecnologia da saúde, há uma grande lacuna que separa avanços tecnicamente atingíveis na saúde e o que os indivíduos e as populações de fato conquistam.^29^ O status financeiro é um fator relevante no manejo da doença, relacionado ao conhecimento funcional da saúde, aderência e autocuidado. Os autores enfatizam que quanto menor o nível socioeconômico, menor a taxa de aderência ao tratamento (com medicamentos ou não).^30^

Na Europa, estima-se que o custo anual do tratamento da IC seja de 3,7% do Fundo Nacional da Saúde, com a expectativa de dobrar esta quantia nos próximos 20 anos. Os custos com medicamentos compõem menos de 5% dos recursos gastos em tratamento de pessoas com IC, enquanto 79% está relacionado a despesas com internação.^31^

No Brasil, a IC tem um custo alto. Kaufman et al.^32^ demonstraram que, entre 2001 e 2012, a duração média da internação de pacientes com IC foi de 5,8 dias, em 2001, e 6,6 dias, em 2012; a taxa de mortalidade estava em crescimento, começando em 6,6%, em 2001, e atingindo 9,5%, em 2012 (aumento de 46,1%). O custo médio da autorização para internação aumentou de R$ 519,54, em 2001, para R$ 1.2019,56, em 2012 (aumento de 132,8%). Uma pesquisa que buscou descrever o número de hospitalizações por IC nas regiões brasileiras em 2017 e o impacto dessas internações nos custos hospitalares mostrou que, só em 2017, a quantia de R$ 339.719.216,50 foi gasta.^28^

Em um estudo recente, Stevens et al.^33^ analisaram os custos associados ao tratamento, perda de produtividade devido à redução da empregabilidade, custos de promover cuidado formal e informal e perda de bem-estar relacionada às condições de quatro grandes doenças cardíacas no Brasil. Os autores descobriram que os custos com IC foram de R$22,1 bilhões (6,8 bilhões de dólares) e que os pacientes tinham perda significativa de produtividade.

O alto custo associado à assistência em IC sugere que novos estudos sejam desenvolvidos para entender melhor as escolhas dos pacientes, para que os profissionais possam estar mais preparados para ajudá-los a administrar suas medicações, influenciar comportamentos diários e incentivar a tomada de decisões saudáveis. Conhecer a situação financeira e entender a economia da cidade pode promover abordagens adicionais para melhorar o prognóstico da doença.

Vale a pena mencionar que cidades com baixo risco de mortalidade por conta da doença precisam ser analisadas com cuidado. A área demonstrada como fator protetivo para mortalidade da doença pode ser resultado de subnotificação. Além disso, a migração de pacientes com IC para regiões vizinhas por conta de melhores oportunidades de tratamento pode dificultar a interpretação do estudo.

Algumas limitações do estudo devem ser relatadas. Primeiro, é um estudo ecológico, então a análise do nível agregado dos dados não pode estabelecer relações entre variáveis no nível individual. Além disso, já que nossos dados derivaram de uma base de dados nacional, é possível que haja subnotificação, e a taxa de mortalidade hospitalar pode ser subestimada. Essas limitações podem trazer uma melhor abordagem aos resultados. Porém, a precisão da análise, a quantidade de dados e o período de tempo analisado (longo) promovem segurança à evidência gerada neste estudo.

## Conclusões

A mortalidade por IC seguiu um padrão espacial no período analisado. A notificação de casos iniciou-se nas cidades do sul do Brasil e, com o passar dos anos, um padrão crescente de mortalidade foi observado em outras regiões, com ênfase no sudeste e no nordeste.

A análise espacial contribuiu para mostrar o cenário da mortalidade por IC nas cidades brasileiras. As áreas geográficas destacadas estão mais suscetíveis à mortalidade, o que requer ações específicas para prevenir a doença e promover a saúde. Assim, este estudo pode guiar ações com foco em melhorar a qualidade do cuidado clínico oferecido em cidades mais afetadas pela IC.
