# Supplementary material for: Distribuição Espacial de Mortalidade por Insuficiência Cardíaca no Brasil, 1996-2017
Source: Arq Bras Cardiol. 2021 Nov 17;118(1):41–51. [Article in Portuguese] doi: 10.36660/abc.20201325 (PMC8959057; doi:10.36660/abc.20201325)
Supplement: Translation

# Translation: Spatial Distribution of Mortality for Heart Failure in Brazil, 1996 – 2017

**Authors:** Virna Ribeiro Feitosa Cestari, Thiago Santos Garces, George Jó Bezerra Sousa, Thatiana Araújo Maranhão, João David Souza, Maria Lúcia Duarte Pereira, Vera Lúcia Mendes de Paula Pessoa, João Tobias Lima Sales, Raquel Sampaio Florêncio, Lorena Campos de Souza, Glauber Gean de Vasconcelos, Maria Gyslane Vasconcelos Sobral, Lara Lídia Ventura Damasceno, Thereza Maria Magalhães Moreira

**Affiliations:** 1 Brazil Universidade Estadual do Ceará - Programa de Pós-Graduação Cuidados Clínicos em Enfermagem e Saúde, Fortaleza, CE – Brazil; 2 Brazil Universidade Estadual do Piauí – Enfermagem, Teresina, Piauí – Brazil; 3 Brazil Hospital de Messejana Dr. Carlos Alberto Studart Gomes, Fortaleza, CE – Brazil; 4 Brazil Universidade Estadual do Ceará - Centro de Educação, Fortaleza, CE – Brazil

**Keywords:** Heart Failure, Spatial Analysis, Ecological Studies, Epidemiology

## Abstract

**Background:**

Heart failure (HF) is a leading cause of mortality and morbidity worldwide, and is associated with the high use of resources and healthcare costs. In Brazil, the HF prevalence is around 2 million patients, and its incidence is of approximately 240,000 new cases per year.

**Objective:**

The present investigation aimed to analyze the spatiotemporal trend of mortality caused by HF in Brazil, from 1996 to 2017.

**Methods:**

This is an ecological study developed with secondary data on HF mortality in Brazil. During the period, 1,242,014 cases of death caused by heart failure were analyzed. The existence of spatial autocorrelation of cases was calculated using the Global Moran Index (GMI) and, when significant, the Local Moran Index, considering p<0.05. The relative risk of the clusters was calculated.

**Results:**

The mortality rate due to HF was diversified in all Brazilian regions, with an emphasis in the South, Southeast, and Northeast. The GMI indicated positive spatial autocorrelation (p=0.01) in all periods. Municipalities located in the South, Southeast, Northeast, and Midwest showed a higher Relative Risk for mortality from HF, and most municipalities in the North were classified as a protective factor against this cause of death.

**Conclusions:**

The study showed a decline in mortality rates across the national territory. The highest concentration of mortality rates is in the North and Northeast regions, highlighting priority vulnerable areas in the planning and controlling strategies of health services.

## Introduction

Heart Failure (HF) is a leading cause of mortality and morbidity worldwide, and is associated with the high use of resources and healthcare costs. In the United States, the estimated number of HF patients aged ≥ 20 years increased from 5.7 million, in 2009-2012, to 6.2 million in 2013-2016,^1 , 2^ and is estimated to affect 26 million people around the world. Its prevalence has been increasing fast due to the aging of the population and better care and treatment.^3 , 4^

In Brazil, the prevalence of HF is of around 2 million patients, and its incidence is of approximately 240,000 new cases per year. This country has the largest universal health system in the world; besides, it is characterized by intense racial admixture, social inequalities, and cultural traditions that may affect the natural history of HF.^5^ It was observed that morbidity and mortality rates related to HF in Brazil are much higher than those observed in developed countries, even when adjusting for region, several hospital beds, and type of institution.^6^

Multiple regions of Brazil have shown a high variation in the quality of care addressed to cardiovascular conditions of high economic burden, such as HF. These registries remain suboptimal, and the lack of optimal therapies is more critical in the public non-academic institutions of the poorest regions of Brazil.^7^

Mortality due to HF is constantly associated with individual, social, economic, and health service indicators.^8^ In this regard, it is relevant to have an in-depth understanding of the spatial distribution of mortality caused by HF to increased knowledge about the areas of greatest vulnerability. The spatial analysis of HF mortality cases throughout the national territory is fundamental for the identification of specific demands and priority locations and, thus, for the planning of interventions that can reduce mortality caused by this disease.

There are no national and international studies about the distribution and spatial analysis of HF mortality rates in the Brazilian territory, denoting an existing knowledge gap.

## Objective

The present investigation aimed to analyze the spatiotemporal trend of mortality from HF in Brazil, from 1996 to 2017.

## Methods

### Design

It is an ecological study that included all regions and municipalities of Brazil as units of analysis. Brazil has a population of 211,389,487 inhabitants distributed in 5,570 municipalities, according to the Brazilian Institute of Geography and Statistics (IBGE) (2020).

The country is divided in five major regions, subdivided into 26 states and the Federal District: Seven states in the North – Acre (AC), Amapá (AP), Amazonas (AM), Pará (PA), Rondônia (RO), Roraima (RR), and Tocantins (TO); nine states in the Northeast – Alagoas (AL), Bahia (BA), Ceará (CE), Maranhão (MA), Paraíba (PB), Pernambuco (PE), Piauí (PI), Rio Grande do Norte (RN), and Sergipe (SE); four states in the Southeast – Espírito Santo (ES), Minas Gerais (MG), Rio de Janeiro (RJ), and São Paulo (SP); three states in the South – Paraná (PR), Rio Grande do Sul (RS), and Santa Catarina (SC); three states in the Midwest – Goiás (GO), Mato Grosso (MT), Mato Grosso do Sul (MS); and the Federal District (DF) ( Figure 1 ).

**Figure 1:** – Brazilian Regions and States.

### Setting

The data were obtained from the website of the Unified Health System (DATASUS), in the sections Vital Statistics (mortality – 1996 to 2017, by ICD-10) and Health Indicators and Pactuation (www.datasus.com.br). These data were collected in July 2019. The population used to calculate the mortality rate per 100 thousand inhabitants and the digital cartographic base were obtained from IBGE.

### Inclusion criteria

All secondary HF mortality data across the country were used (classification I50 in the ICD-10), diagnosed from January 1996 to December 2017, and recorded in the Mortality Information System (MIS).

### Analysis

After identifying the number of deaths from HF in the municipalities, the mortality rate was calculated. We used, as numerator, the total number of deaths identified each year by the resident population of the same year, for each of the observed years, from 1996 to 2017. Then, the resulting value was multiplied by the constant 100 thousand.

We analyzed the temporal pattern of HF mortality in Brazil in general and by regions. The data organized in tables, in Microsoft Excel, were transferred to the Joinpoint Regression software, version 4.6.0.0. With this software, it is possible to make a time series analysis of the most diverse health problems. It also performs segmented linear analysis (also known as analysis by inflection points, or joinpoints), calculating the logarithmic transformation of the values.

The software works with the null hypothesis that a segment of one line can explain possible variations over the years, and that the alternative to this process would be the inclusion of inflection points in the period, with a consequent change in the slope of the line segment. The program calculates the annual percentage change (APC) with a 95% confidence interval (95%CI).

The interpretation given to these values is that a positive APC indicates an increasing trend, and a negative one indicates a decreasing trend. Furthermore, at the end of the period, it is possible to calculate how the changes during the periods behaved using the average annual percentage change (AAPC), which has an interpretation similar to APC, but, in this case, aimed at the entire period.

Following this principle, this study worked with the nullity hypothesis that the simple linear trend could express the variation in the mortality rate due to HF in Brazil and that, as an alternative, points should be included in the model with the change in line segments. A significance level of 5% was established to test the hypotheses of APC and AAPC of the series. For both cases, it was considered as significant when the model had p<0.05 or 95%CI, entirely positive or negative.

The analyzed years were divided in four periods: A (1996-2001), B (2002-2007), C (2008-2012), and D (2013-2017). These crude rates were smoothed through the local empirical Bayesian method to reduce the instability caused by the crude rates. This method considers not only the number of deaths by municipality, but weighs it with the neighboring municipalities through a spatial proximity matrix. To calculate this matrix, the contiguity criterion was used, considering value 1 to the neighboring municipalities and 0 to the non-neighbors.^9^

After a descriptive spatial analysis, the presence of global spatial dependence was evaluated using the Global Moran Index (GMI) on crude indicators. The method identifies spatial autocorrelation and ranges from -1 to +1, in which values close to zero indicate the absence of spatial dependence, considering p<0.05 as significant. Additionally, the Local Index of Spatial Association (LISA) was assessed by the Local Moran Index, which checks the value of the municipality and its neighbors for the identification of spatial patterns of the studied phenomenon.^10^

The Local Moran Index generates the Moran’s scattering diagram, which consists of four quadrants: high-high (municipalities with high rates and surrounded by those with high rates), low-low (municipalities with low rates surrounded by those with low rates), high-low (municipalities with high rates surrounded by those with low rates) and low-high (municipalities with low rates surrounded by those with high rates), considering p<0.05 as significant. The high-high and low-low categories represent areas of agreement, and the high-low and low-high categories indicate epidemiological transition areas.^10^

The relative risk of clusters was calculated using the spatial scanning technique, called the Scan statistic. It was used to identify risk and protection areas for HF mortality. For that end, the relative risk (RR) of each municipality for mortality was calculated, and the presence of purely spatial death clusters was identified.

Statistical analysis was performed by using Stata software, version 12.0. Joinpoint regressions were performed by the Joinpoint Regression software, version 4.7.0.0. Spatial statistics were performed with TerraView 4.2.2. The purely spatial scan analysis was performed with the aid of SaTScan 9.6 software. All maps were created using QGIS 2.4.17.

### Ethical considerations

This study dismissed previous approval from Ethics Research Committees since the database was freely available on the internet by the Brazilian Government. It is important to emphasize that no identification information, such as name or address, was present in the database. Even though the researchers did not need previous approval, they declared their ethical commitment in handling data, analysis, and publication, as established by the National Research Council Resolution 466/12. The research was reported via strengthening the reporting of observational studies in epidemiology (STROBE).

## Results

During the 21 year-period, 1,242,014 deaths by HF were notified. The temporal trend analysis results showed a significant decrease of 2.3% (95%CI: -2.3 – -2.7) in the mortality rate in all Brazilian territories. By considering the continental dimensions of Brazil, it is necessary to investigate such trends in regional areas. Four of the five national Regions showed a significant reduction in mortality rates, highlighting a minimal reduction in mortality rates in the Northeast (AAPC: -0.7; 95%CI: -0.9 – -0.4). On the other hand, the South increased its mortality rate by 2.9% (95%CI: 2.0 – 3.9) ( Table 1 ).

**Table 1:** – Temporal trends of heart failure mortality rates in Brazil and its regions. Fortaleza, CE, Brazil, 2019

Country Regions	*APC1	^‡^IP	*APC2	^‡^IP	^*^APC3	^‡^IP	†AAPC
			
	(§ 95% CI)		(§ 95% CI)		(§ 95% CI)		(§ 95% CI)
Brazil	-6.3 **//** (-7.7; -1.8)	2001	-1.1 **//** (-1.3; -0.7)	2017			-2.3 **//** (-2.3; -2.7)
North	-1.3 (-11.3; 2.4)	2017					-1.3 **//** (-1.7; -0.9)
Northeast	- 0.7 **//** (-7.1; -1.5)	2017	9.4 **//** (6.6; 12.3)	2011	-2.8 (-4.2; -1.4)		-0.7 **//** (-0.9; -0.4)
Southeast	-7.7 (-8.7; -6.7)	2002	-0.8 (-1.2; -0.3)	2014	-2.4 **//** ( -0.9; -5.8)	2017	-4.0 **//** (-5.5; -2.4)
Midwest	-6.2 **//** (-10.2; -2.1)	2000	-2.3 **//** (-3.0; -1.7)	2015	-3.3 ( -11.5; 3.8)	2017	-4.0 **//** (-5.5; -2.4)
South	-7.9 **//** ( -13.0; -2.5)	1998	37 **//** (28.9; 45.5)	2001	-1.1 **//** (-1.3; -0.8)	2017	2.9 **//** (2.0; 3.9)

*APC: Annual Percentage Change; **†** AAPC: Average Annual Percent Change; **‡** IP: Inflection Point; **§** 95%CI: 95% Confidence Interval; **//** p = < 0.05

Regarding the spatial analysis of the 5,570 municipalities, the maps in Figure 2 demonstrate that crude mortality rates varied from 0 to 200 deaths by 100 thousand inhabitants. In the red areas, it is possible to identify, in period A (1996-2001), that high mortality rates were focused on municipalities of the South and the Southeast. Periods B (2002-2007) and C (2008-2012) demonstrate that, throughout the years, rates spread to the Northeast and the Midwest, but kept high concentrations in the South (similar results to the time series analysis). In the last years, as demonstrated in period D (2013-2017), it is possible to highlight that the municipalities in the North did not present high mortality rates.

**Figure 2:** – Maps of crude mortality rates by heart failure in Brazil, in periods A (1996-2001), B (2002-2007), C (2008-2012), D (2013-2017), per municipality and per 100 thousand inhabitants. Fortaleza, CE, Brazil, 2019.

In Figure 3 , the rates were smoothed by the empirical Bayesian method and demonstrated that, in period A (1996-2001), mortality was mostly concentrated in municipalities in the South; however, they varied throughout the years. Period B (2002-2007) showed that the Southeast presented high mortality rates, particularly in municipalities located in the states of São Paulo and Minas Gerais. In periods C (2008-2012) and D (2013-2017), it was possible to identify a high death concentration in the Northeast, predominately in Piauí, Bahia, Rio Grande do Norte, and Ceará.

**Figure 3:** – Maps of mortality rates due to heart failure in Brazil smoothed by the empirical Bayesian method in periods A (1996-2001), B (2002-2007), C (2008-2012), and D (2013-2017), per municipality and per 100 thousand inhabitants. Fortaleza, CE, Brazil, 2019.

Regarding autocorrelation spatial analysis, the GMI of period A (1996-2001) was 0.41 (p=0.01); in period B (2002-2007), it was 0.28 (p=0.01); period C (2008-2012) it was 0.29 (p=0.01); and GMI in period D (2013-2017) was 0. 31 (p=0.01). These results indicate positive spatial autocorrelation.

Since spatial dependency was verified in all study areas by GMI, we used the Local Moran Index (LMI). The spatial clusters corroborate the results in the empirical Bayesian local maps in all periods. They confirmed that high-highs are more concentrated in the South and spread to Northeast municipalities over the years. The low-low was concentrated in cities of the North and Midwest regions ( Figure 4 ).

**Figure 4:** – Maps of Moran’s spatial autocorrelation of mortality by heart failure in Brazil in periods A (1996-2001), B (2002-2007), C (2008-2012), and D (2013-2017). Fortaleza, CE, Brazil, 2019.

In Figure 5 , scan spatial statistics confirmed other analyses and identified that the greater risk of CI mortality was in the South in period A (1996-2001). This pattern advanced in other periods, being also possible to find high RR in the Midwest.

**Figure 5:** – Relative risk of all Brazilian municipalities to heart failure mortality in periods A (1996-2001), B (2002-2007), C (2008-2012), and D (2013-2017). Fortaleza, CE, Brazil, 2019.

Nevertheless, the last period also demonstrated higher risks in the North and the Northeast. In the last period (2013-2017), almost all cities in the South, Southeast, Northeast, and Midwest had some risk of mortality from heart failure. In the North, the cities in the states of Pará and Amazonas had little risk of mortality, with RR<1 (in blue); only a few cities of Roraima and Acre had clusters with RR>15 (in red).

## Discussion

This is the first study to assess the spatial distribution of HF mortality in Brazil. Initially, the municipalities in the South had a higher mortality rate. This notification pattern characterized operational contexts of “epidemiological silence” in some areas until 2001. Since 2002, mortality rates have increased in municipalities in the Southeast, Northeast, and the Midwest. Municipalities in the North, on the other hand, have shown low mortality rates, revealing the underreporting of cases.

It is important to observe that the municipalities in Brazilian regions with the highest mortality rates are the largest in population size and have more elderly people. National and international surveys have shown that HF mainly affects individuals aged more than 65 years.^11 , 12^ The increasing prevalence of HF is directly related to the most common comorbidities in aging.^13^ Besides, advanced age is a risk factor for self-care, poor treatment adherence, and disease management.^14 - 16^

The disease is prevalent among the elderly, but a high percentage of the disease is also perceived among adults.^17^ In an analysis of specific time trends for age, sex, and race in HF-related morbidity and mortality rates between the years of 1991 and 2015, in the United States, Vasan, Zuo & Kalesan^18^ observed an increase in hospitalization rates in people with the disease in the age group of 30 to 49 years. This fact may be related to an inadequate lifestyle, exposing individuals to numerous risk factors for the disease, such as smoking, alcohol consumption, physical inactivity, and comorbidities, with emphasis on hypertension and diabetes.

Poffo et al.^19^ attributed the number of HF cases in adults to the earlier manifestation of heart disease, less effective treatment of diseases that lead to HF, and non-adherence to the recommended treatment. Thus, prevention stands out as an important weapon to fight against HF.^17 , 18 , 20^ The high mortality rate in municipalities in the southern region may be associated with the race/ethnicity of people affected by HF, with a higher prevalence of white individuals. While the disease prevails in this population, survival is higher among black people (7.5% vs. 2.2%; p = 0.01),^19^ Hispanics, and Asians.^21^

The reason for such a health disparity is complex. The effect of race on the prognosis of HF is uncertain because different studies have revealed divergent results. Race showed no association with the occurrence of complications and hospital readmissions among Australians with HF.^22^ Husaini et al.,^23^ in a study comparing race/ethnicity to hospitalization rates, found that hospitalization rates for black individuals remained higher compared to whites and Hispanics.

In a North American study, in a total of 7,878 patients with HF, 35.8% were black. Among them, diseases such as hypertension, diabetes, and cerebrovascular diseases were prevalent; however, the mortality rate was lower when compared to white individuals.^24^ Goyal et al.^25^ analyzed 1,889,608 hospitalizations of patients with HF and found out that other comorbidities, such as anemia and chronic kidney disease, were more common among blacks, but this group presented lower in-hospital mortality when compared to whites.

The divergence in the literature reinforces the need for further research on the association between race/ethnicity and mortality rate due to HF, as well as the main causes of readmission. Such findings may assist in the implementation of preventive programs for hypertension and diabetes among minorities, which can reduce subsequent hospitalization for HF in these patients.

The change in the epidemiological patterns of the disease in the country has also been generated as a result of coverage by health services. Health care in Brazil is guided by a clear definition of territories and the people under the responsibility of each health team. The improvement of access, quality, and infrastructure of health care and coverage of Brazilian municipalities is a concern of health managers.

It is necessary to keep in mind that there are regional differences between the conditions of access to health institutions and the care provided by Brazil. Differences in supply can be observed within the same municipality, between municipalities and between states of the federation, which results in the maintenance of an uneven distribution pattern of supply and access to services.^26^

The complexity of HF requires knowledge from different professionals who work at different levels of health care. Therefore, it is important to articulate these levels to promote health, quality of care, and user satisfaction. Health providers and patients must take on some responsibility that extends beyond mechanistic or traditional care. It is necessary to empower and enable the individual to protect oneself from situations of vulnerability, thus reducing regional disparities.

The increase in mortality rates due to HF in the municipalities of the North and Northeast can be explained by the low quality of life and stressors, which are the main agents for higher increase in hospitalization. Hospitalizations occur not only due to HF, but also other comorbidities.^19 , 27^

The Brazilian regions with the highest number of hospitalizations in 2017 were the Southeast, followed by the South and Northeast, and, in the third place, the Midwest and the North.^28^ Isolated initiatives suggest the existence of significant regional differences in several characteristics of patients hospitalized with HF in Brazil,^17^ although these comparisons are methodologically limited by often divergent designs and inclusion criteria.

Despite major advances in health care science and technology, a large gap separates technically achievable advances in health from what individuals and populations actually achieve.^29^ Financial status is a relevant factor in the management of the disease, related to functional health literacy, adherence, and self-care behaviors. The authors emphasize that the lower the socioeconomic level, the lower the rate of treatment adherence (medicated or non-medicated).^30^

In Europe, it is estimated that the annual cost of HF treatment is 3.7% of the National Health Fund, with the expectation of doubling the amount in the next 20 years. Medication costs make up for less than 5% of the resources spent on treating people with HF, whereas 79% is related to expenses with hospitalization.^31^

In Brazil, HF has a high financial cost. Kaufman et al.^32^ revealed that, in the period from 2001 to 2012, the average length of stay for patients with HF was 5.8 days, in 2001, to 6.6 days in 2012; the mortality rate was on the rise, starting from 6.6%, in 2001, and reaching 9.5% in 2012 (a 46.1% increase). The average cost of authorization for hospitalization increased from R$ 519.54, in 2001, to R$ 1,209.56 in 2012 (an increase of 132.8%). Research that sought to describe the number of hospitalizations for HF in Brazilian regions in 2017 and the impact of these hospitalizations on hospital costs showed that, in 2017 alone, the amount of R$ 339,719,216.50 was spent.^28^

In a recent study, Stevens et al.^33^ analyzed the costs associated with treatment, lost productivity due to reduced employment, the costs of providing formal and informal care, and loss of well-being related to the conditions of four major heart diseases in Brazil. The authors found that the costs with HF were R$22.1 billion (USD 6.8 billion) and patients had significant loss in productivity.

The high cost associated with care in HF suggests that new studies are developed to better understand the patients’ choices, so that professionals can be more prepared to help them administer their medications, influence daily behaviors, and encourage taking healthy decisions. Knowing the financial situation and understanding the municipality’s economy can provide additional approaches to improve the prognosis of the disease.

It is worth mentioning that cities at low risk of mortality from the disease need to be analyzed with caution. The area demonstrated as a protective factor for mortality from the disease may be the result of underreporting. Moreover, the migration of patients with HF to neighboring regions due to better opportunities for treatment may make it difficult to interpret the study.

Some limitations regarding this study should be reported. First, it is an ecological study, so the analysis of aggregated level patient data cannot establish relationships between individual-level variables. Furthermore, since our data derived from a national database, it is possible that there is underreporting, and the hospital mortality rate may be underestimated. These limitations may better address the results. On the other hand, the curacy of the analysis, the amount of data and the long analyzed time period provide safety to the evidence generated in this study.

## Conclusions

HF mortality followed a spatial pattern in the analyzed period. The notification of cases was initiated in the municipalities of the South of Brazil and, over the years, an increasing pattern in mortality was observed in other regions, with emphasis on the Southeast and the Northeast.

Spatial analysis contributed to show the HF mortality scenario in Brazilian municipalities. The highlighted geographic areas are most susceptible to mortality, requiring specific actions to prevent the disease and promote heath. Therefore, this study may guide actions aimed at improving the quality of clinical care provided in the cities most affected by HF.
